# Flexible or rigid control of eating scale: development and validation of the FORCES in women

**DOI:** 10.1186/s12966-025-01746-3

**Published:** 2025-04-11

**Authors:** Kate Nicholls, Lenny R. Vartanian, Kate Faasse, Jennifer S. Mills

**Affiliations:** 1 School of Psychology, Faculty of Science, UNSW Sydney, High Street Kensington, Sydney, NSW 2052 Australia; 2 Department of Psychology, Faculty of Health, York University, Toronto, Canada

**Keywords:** Dietary restraint, Disinhibition, Rigid control, Flexible control, Scale development

## Abstract

**Background:**

Many dieters show a pattern of disinhibited eating following a diet violation, and it has been proposed that the nature of their dietary restraint (i.e., whether they are rigid or flexible in their pursuit of dietary control) could prove beneficial in explaining variability in the occurrence of disinhibited eating. However, existing measures of rigid and flexible control do not adequately separate these two styles of dietary restraint.

**Method:**

The current studies aim to develop a new scale that more clearly differentiates the constructs of rigid and flexible control of eating. Exploratory and confirmatory factor analysis were used to develop and validate the new scale across three distinct samples of women (total *N* = 1048).

**Results:**

Factor analysis identified five total factors: three relating to the rigid control of food intake (Strict Behaviours, Negative Emotions, and Worry), and two relating to the flexible control of food intake (Flexible Beliefs and Positive Emotions). The Flexible or Rigid Control of Eating Scale (FORCES) had good internal consistency, a reliable factor structure that replicated across the three samples of women and provided a clear separation between rigid and flexible control.

**Conclusions:**

The FORCES may allow researchers to explain why some dieters are more likely to engage in disinhibited eating than are others and can be a beneficial step toward addressing the negative consequences of maladaptive dieting behaviour.

**Supplementary Information:**

The online version contains supplementary material available at 10.1186/s12966-025-01746-3.

## Background

Restrained eaters (chronic dieters) are a population of individuals who attempt to limit or restrict their food intake, often for the purpose of controlling their weight [1]. Dieting behaviour is consistently shown to be more prevalent among women than men, with estimates suggesting that up to 65% of women are engaging in some form of dieting, compared to 40% of men [2]. Frequent dieting behaviour is associated with negative psychological outcomes, including body dissatisfaction and a pervasive desire to be thin [3]. In one sample of American women aged 25–89, almost 90% identified that their current body size was larger than their ideal body size [4]. It is clear that dieting and the associated psychological consequences are an essential area for further research and intervention, particularly within female populations.

Despite intending to restrict their food intake, many dieters cycle between periods of restriction and periods of indulgence or disinhibited eating [5]. Over the last five decades, research has demonstrated that a variety of factors can cause restrained eaters to abandon their restriction and engage in disinhibited eating. The consumption of an initial high-calorie or diet-breaking food, commonly referred to as a preload [6, 7], the experience of negative and positive mood states [8–10], a high cognitive load [11], and the consumption of alcohol [12] have all been shown to lead dieters—but not non-dieters—to engage in overindulgence when they are subsequently presented with highly-palatable food to eat. The range of factors that have been identified as disinhibitors suggest that restrained eaters are highly susceptible to disinhibited eating.

One explanation that has been proposed to account for disinhibited eating is the ‘what the hell’ effect [13]. This explanation posits that, when a dieter perceives their diet to be broken or threatened, a motivational shift occurs. Rather than compensating for the perceived diet transgression and returning to their intended diet, the dieter abandons their immediate commitment to restrict their food intake. This motivational shift leads the dieter to engage in a period of overindulgence, in which they are likely to consume foods that they would typically not allow themselves to eat.

Although numerous studies have shown disinhibition among restrained eaters as a group, there are likely individual differences in the degree to which restrained eaters engage in disinhibited eating. One distinction that has been proposed to explain this individual difference is the notion of rigid vs. flexible control. Restrained eating is broadly defined as the *intention* to restrict one’s food intake, but individuals may differ in their approach to restricting their consumption [14]. Those who employ a rigid dieting strategy are likely to take a dichotomous approach to dieting, believing that the diet is either intact or broken. These individuals are likely to implement a strict upper limit for their food intake and believe that some foods are ‘good’ or ‘healthy’ and acceptable to consume whereas others are ‘bad’ or ‘unhealthy’ and should be avoided entirely [14, 15]. This strict, rigid approach to dieting likely means that if a ‘bad’ or ‘unhealthy’ food is consumed, or if the individual surpasses their intake limit, then they will experience the ‘what the hell’ effect and engage in disinhibited eating [13]. Flexible dietary restraint, in contrast, represents a less strict approach to dieting, whereby restrained eaters employ a form of self-regulation, making allowances for deviations from the intended diet in social settings (e.g., when social norms include indulgent eating), or in response to hunger levels or taste preferences [14]. Flexible dieters acknowledge that foods that they normally try to avoid are acceptable in small amounts and/or at certain occasions. Incorporating flexibility into the dieting approach may make the dieter less likely to adopt a ‘what the hell’ attitude when their diet is broken, and therefore less likely to engage in disinhibited eating.

The existing Rigid and Flexible Control scales were developed to measure these different dieting strategies, reflecting high and low susceptibility to disinhibition, respectively [15]. The Rigid and Flexible self-report scales were initially created using the Restraint and Disinhibition subscales of the Three-Factor Eating Questionnaire (TFEQ) [16], a commonly used measure of dietary restraint. Items from the TFEQ-Restraint subscale that were associated with high scores on the TFEQ-Disinhibition subscale were allocated to the measure of Rigid Control, whereas items associated with low scores on the TFEQ-Disinhibition subscale were allocated to the measure of Flexible Control [15]. In subsequent research, new items were developed and added to the Rigid and Flexible Control scales [17].

Several studies have provided evidence for the utility of separating dietary restraint into rigid and flexible control. For example, flexible control has been shown to predict the amount of food eaten by participants following the consumption of a high-calorie preload, with those scoring higher on flexible control subsequently consuming less food than those scoring lower on flexible control [18]. Individuals who engage in flexible control appear to be able to regulate and even reduce their food intake after the consumption of a high-calorie food. This result could suggest that the disinhibiting effect of a preload may be limited to rigid dieters only. However, this study did not measure rigid control, so this suggestion remains speculative. Other research has provided evidence that dieters who were more rigid in their approach to eating engaged in different forms of weight management than did those who were more flexible in their approach [17]. Greater rigid control was associated with unhealthy weight control practices, such as the use of diuretics, laxatives, appetite suppressants and vomiting, whereas more flexible dieting was associated with physical activity and body building [17]. The use of strategies such as physical activity and body building may suggest that flexible dieting is associated with a more long-term focused approach to weight management, compared to the ‘quick-fix’ approaches associated with rigid dieting. If that is the case, then flexible dieting practices may be more likely to result in successful weight management than would rigid control. Indeed, studies have shown that higher scores on the Flexible Control scale were associated with more successful long-term weight management, relative to lower scores for flexible control [19–22]. Among participants attempting to lose weight, greater flexible control of one’s eating was associated with a lower body mass index (BMI), whereas more rigid control was associated with a higher BMI [17, 21], suggesting that rigid control may lead to weight gain [23]. Alternatively, individuals who experience weight gain may engage in rigid dietary practices in an attempt to lose weight or to prevent further weight gain, whereas individuals who have a more stable weight may be more open to engaging in flexible dietary control.

Despite the clear value of understanding the heterogeneity of dietary restraint, there are a number of issues with the existing measures of rigid and flexible control, particularly regarding the lack of clear separation between the two scales. Many studies have noted a strong *positive* correlation between scores on the rigid and flexible control measures, suggesting that the scales do not adequately separate the two concepts [24–26]. In addition, the two subscales are often associated with various outcomes in the same direction. The measures of rigid and flexible control have both been shown to correlate positively with disordered eating symptoms, body image concerns, body shape concerns, weighing frequency, and perceived weight cycling, and negatively correlate with body appreciation [24, 25, 27]. One study even identified that dichotomous thinking, which *should* be more reflective of a rigid style of restraint than a flexible style, was positively correlated with both rigid and flexible control [25]. It is difficult to examine whether flexible control is associated with more consistent long-term dietary restraint when scores on the current measure are so closely related to scores on the measure of rigid control [26]. The lack of clear separation between the two scales, and the positive correlations detected between them, are perhaps not surprising given that several of the items included on the two scales overlap conceptually. For example, one item on the rigid control scale asks whether participants “count calories as a conscious means of controlling my weight”, and an item on the flexible control scale asks if the following statement is true “when I have eaten my quota of calories, I am usually good about not eating any more” [17]. Both items refer to an underlying concept of monitoring calorie consumption, with a fixed upper limit for the amount that is acceptable to consume. It is not clear how these two items would help to differentiate between a rigid and flexible approach to dieting, representing one example of how the content validity of the existing measures could be improved.

Research has also produced inconsistent results when attempting to replicate previous findings of the relationship between rigid and flexible control and other relevant variables. In terms of eating-related psychopathologies, one study found that neither rigid nor flexible control scores had a significant association with objective or subjective bulimic episodes, objective overeating, eating concerns, or disinhibition scores [28]. Researchers have also been unable to fully replicate the differential associations between rigid and flexible control and BMI in community samples [26, 29]. In one study, the association between rigid control and high BMI and flexible control and low BMI was replicated amongst women but not men [29]. Another study found a positive correlation between rigid control and BMI, but no significant relationship between BMI and flexible control [26]. This failure to replicate previous findings that were used to support the convergent validity of the Rigid and Flexible Control scales raises questions about the validity of these measures.

Given the potential theoretical and practical benefits of differentiating rigid and flexible dieting strategies, and the weaknesses of the measures as reviewed above, the current studies aim to develop a new scale to assess the rigid and flexible control of eating. The new scale is intended to address the limitations of the existing measures by providing a clearer separation between the two concepts. A set of three studies was conducted to develop and validate the new scale using female samples. Study 1 involved item development and exploratory factor analysis to identify relevant factors, Study 2 involved confirmatory factor analysis to ensure the factor structure of the scale was reliable across samples, and Study 3 involved validating the new scale with existing measures of relevant, related concepts. A more reliable and valid measure of the rigid and flexible control of eating could be a useful tool for future research to examine why some restrained eaters are more susceptible to disinhibited eating than are others.

## Study 1

### Methods

#### Participants

Participants were recruited via Prolific, an online research platform where participants can complete online surveys for monetary reimbursement. Surveys are posted on the Prolific platform, and participants can select which surveys they are interested in completing. A total of 450 participants were recruited for this study. This sample size was selected because there were 43 initial items created for the new measure, and researchers typically recommend a 10:1 participant-to-item ratio for an exploratory factor analysis [30]. Participants were paid 0.9GBP for completing the 6-min survey. Eligibility was limited to participants who resided in Australia, New Zealand, Canada, the United States, or the United Kingdom, and who identified as female in the Prolific pre-screen. Eligibility was limited to these countries due to the existence of a similar societal pressure across Western nations for women to strive towards a thin-body ideal. Female participants were recruited based on the understanding that dieting is more common among women than men [31], the psychological consequences of dieting are particularly pervasive among women [4], and because most previous research on dietary restraint has focused on women [6, 8–12, 32–36]. Furthermore, research that has been conducted to develop, validate, support, and critique the original measures of rigid and flexible control has recruited samples that are either entirely or predominantly female [15, 19–22, 24, 25, 27–29, 37]. Thus, to ensure that the newly developed measure can be appropriately compared to the existing measures, and to understand the influence of rigid and flexible dieting within the population that engages in dieting behaviour most commonly, the current studies also focused on participants who identified as female.

From the sample characteristics provided by participants in the current study, 447 described their gender as female, 2 indicated that they used a different term, and 1 preferred not to respond (no respondents explicitly identified their gender as male, and so all participants were retained for analysis). Participants were asked to complete a captcha verification and a data quality check to assist in identifying any bot responses. All participants completed the verification and data quality check correctly. Participants had a mean age of 38.2 years (*SD* = 12.8, range 19–79), and the mean BMI was 25.96 (*SD* = 7.37, range 9.34–58.69^1^). In terms of ethnicity, 87.6% identified as White, 5.8% as Asian, 3.8% as Black/African American, and 0.9% as Hispanic/Latino(a). The remaining 2% either indicated their ethnicity as ‘other’ or did not respond. When asked if they had ever been diagnosed with a clinical eating disorder, 4.7% indicated that they had, 94.9% had not, and 0.4% preferred not to respond.

#### Materials and procedure

Participants signed up for a study investigating ‘Health Beliefs and Behaviours’ and were informed that they would be asked to complete a series of questionnaires online. After providing electronic consent, participants responded to the newly developed items to assess the rigid and flexible control of eating, along with a series of questions to determine the sample characteristics. This study received ethics approval from the UNSW HREAP-C (File 3654).

##### Flexible or Rigid Control of Eating Scale (FORCES)

The authors (with feedback from experienced researchers in the fields of dietary restraint and health psychology) generated a set of theoretically derived and face valid items to operationalise the distinguishing features of rigid and flexible patterns of dietary restraint. For this purpose, rigid control was defined as having strict rules that dictate one’s food intake, expending significant effort to follow these rules, and experiencing significant distress if rules are broken. Flexible control was defined as having loose guidelines or ideas about how much or which foods one would like to normally eat, dedicating only minimal time and effort to implementing these guidelines, and not being emotionally impacted if guidelines are occasionally ignored. An initial set of 43 items was compiled for the new measure: 28 novel items were created using the definitions of rigid and flexible control described above, and an additional 15 items were adapted from existing measures including the Restraint Scale [38], TFEQ [16], Dutch Eating Behaviour Questionnaire (DEBQ) [39], Intuitive Eating Scale [40], Eating Disorder Examination Questionnaire [41], Dichotomous Thinking in Eating Disorders Scale (DTEDS) [42], and the Obligatory Exercise Questionnaire [43]. Items were created across three broad domains thought to be relevant to the characterisation of the nature of an individual’s restraint, namely: behaviour (e.g., “I follow strict rules about how much I can eat”), effort (e.g., “I spend a lot of time making sure that I am following my diet”), and emotion (e.g., “If I eat something that I would normally not allow myself to eat, I feel like a failure”).

The instructions to participants for the FORCES were as follows: ‘The following questions ask about your eating and dieting behaviours. Please note that, in this context, dieting refers to rules, restrictions, or guidelines you might have about how much or what kinds of foods you allow yourself to eat. This does not include foods that you avoid due to allergies, intolerances, or advice from a health-care professional related to a specific medical condition. Please respond to each item based on how you typically feel and behave’. These instructions were designed to guide participants to focus on dieting rules or restrictions that are self-imposed, rather those that may be motivated by health-related reasons. This distinction is based on existing research which demonstrated that individuals who were motivated to diet or lose weight for health-related reasons (rather than appearance related reasons) scored lower on restraint, disinhibition, body dissatisfaction, body image concerns, and higher on self-esteem [44, 45]. Therefore, health-related reasons for restricting one’s food intake were considered less relevant in the context of examining why some dieters are more susceptible to periods of disinhibited eating than are others. All items were rated on a four-point scale, ranging from 1 (*Not at all True*) to 4 (*Very true*).

##### Sample characteristics

Participants were asked to report their age, gender, and ethnicity. They were also asked to report their current height and weight (to calculate their BMI) and indicate whether they had ever been diagnosed with a clinical eating disorder.

#### Analysis

The original pool of items for the FORCES were subjected to exploratory factor analysis. Principal axis factoring with oblique rotation (direct oblimin) was selected, given that the extracted factors were expected to correlate with one another [46]. The suitability of the data for factor analysis was assessed using Bartlett’s test of sphericity and the Kaiser–Meyer–Olkin (KMO) measure of sampling adequacy [47, 48]. Multiple methods were used to determine the number of factors to extract, including examining the eigenvalues and scree plot, and conducting parallel analysis and the minimum average partial (MAP) test [49–52]. Items were to be retained on the scale if their primary loadings were greater than 0.5, cross loadings were less than 0.2, and item-total correlations were greater than 0.3 [53, 54]. Bivariate correlations were conducted to examine the associations among the factors that emerged. All analyses were conducted using SPSS version 28.0.1.1 (14) [55].

### Results

#### Factor analysis

Most of the initial 43 items were not normally distributed. Normality is beneficial for exploratory factor analysis, but it is not considered an essential assumption that must be met to proceed [46]. There were no missing data for any of the items. One item had insufficient response variation (there were no responses for ‘Very True’), and was therefore removed, leaving 42 items for factor analysis.

Bartlett’s test of sphericity indicated that the data were suitable for factor analysis, with a significant chi-square result (χ^2^(861) = 12,689.87, *p* < 0.001). However, this test can be overly sensitive when the participant to item ratio is greater than 5:1 [30]. Given that the current study had a participant to item ratio of more than 10:1, the KMO measure of sampling adequacy was also used [48]. The KMO measure of sampling adequacy was 0.94, indicating that the data were ‘marvellous’ for factor analysis [56]. Inspection of the eigenvalues suggested a seven-factor solution [52]. However, the scree plot showed a clear point of inflexion at the fourth factor, suggesting a three-factor solution [51]. The parallel analysis method suggested a six-factor solution [50], while the MAP test also suggested a seven-factor solution [49]. Given that two of the four methods suggested a seven-factor solution and that specifying too few factors can result in a loss of important information [46], the seven-factor solution was examined.

One of the factors in this solution was comprised of only two items, both relating to dichotomous thinking about food. Retaining a factor with fewer than three items is not recommended, given that the factor is likely to be unstable in future replications [30, 46]. Another factor, which contained items about food preparation, had no primary loadings above the pre-established cut-off of 0.5 for item retention. These two factors (and the associated six items) were removed, and the factor analysis was re-run on the remaining 36 items, limiting the number of extracted factors to five.

#### Item reduction

Items with poor primary loadings (*n* = 7), high cross loadings (*n* = 2), and poor item-total correlations (*n* = 1) were removed from the scale. This resulted in a five-factor solution containing 26 items. Three of the factors contained three items each, whereas the remaining two factors were comprised of seven and ten items. To ensure that the factors contained a similar number of items, the two larger factors were reduced to a maximum of five items each. Items with the lowest primary loadings (*n* = 4), or a lower primary loading than another similar item (*n* = 3) were removed. The final solution contained 19 items: Factors 1 and 2 contained 5 items, and Factors 3, 4, and 5 each contained 3 items. The principal axis factoring was again repeated to ensure that the final solution maintained the five-factor structure. Table 1 displays the factor loadings for the final solution.

**Table 1:** Pattern matrix for the final five-factor solution of the FORCES with 19 Items

	Factor				
	1	2	3	4	5
Eigenvalues (% variance explained)	2.04 (10.72)	7.13 (37.51)	0.98 (5.15)	1.51 (7.93)	2.40 (12.61)
**Strict Behaviours Subscale** (α = .85, ω = .86)					
*The tendency to engage in specific rigid behaviours relating to the control of food intake*					
I follow strict rules about how much I can eat	**0.777**	-0.008	-0.122	-0.014	-0.010
I pay very close attention to what I eat	**0.733**	-0.016	0.006	-0.075	0.007
I spend a lot of time making sure that I am following my diet	**0.713**	0.098	-0.018	-0.096	-0.004
I check the nutritional content of what I eat	**0.676**	0.005	0.046	0.000	0.039
I keep a detailed record of what I eat	**0.659**	-0.004	-0.035	0.100	-0.047
**Negative Emotions Subscale** (α = .93, ω = .93)					
*The experience of negative emotions following the consumption of something that an individual would normally not allow themselves to eat*					
If I eat something that I would normally not allow myself to eat, I feel like a failure	-0.143	**0.857**	-0.119	-0.013	0.012
If I eat something that I would normally not allow myself to eat, I feel mad at myself	0.044	**0.850**	-0.003	-0.019	0.005
If I eat something that I would normally not allow myself to eat, I feel disappointed in myself	-0.039	**0.836**	-0.095	0.011	0.001
If I eat something that I would normally not allow myself to eat, I feel upset	0.044	**0.827**	0.010	-0.035	-0.036
If I eat something that I would normally not allow myself to eat, I feel tense, irritable, and/or depressed	0.132	**0.777**	0.072	0.012	-0.014
**Worry Subscale** (α = .86, ω = .87)					
*The degree to which an individual is concerned about their ability to maintain control over their food intake*					
I worry that I will lose control over how much I eat	0.017	-0.046	**-0.925**	0.011	-0.036
I worry that I will lose control over what I eat	0.002	0.071	**-0.820**	-0.077	0.044
I feel like food controls me, rather than the other way around	0.106	0.157	**-0.543**	0.039	-0.039
**Flexible Beliefs Subscale** (α = .82, ω = .83)					
*The degree to which an individual believes that any food is acceptable to consume occasionally and/or in small amounts*					
I think that any food is okay to eat occasionally	0.055	-0.023	0.012	**0.960**	-0.066
I think that any food is okay to eat in small amounts	-0.051	0.076	0.072	**0.770**	0.027
It is okay to sometimes eat foods that I would normally not allow myself to eat	-0.039	-0.097	-0.065	**0.571**	0.104
**Positive Emotions Subscale** (α = .84, ω = .84)					
*The experience of positive emotions after eating something that an individual would normally not allow themselves to eat*					
If I eat something that I would normally not allow myself to eat, I feel delighted	0.009	0.017	0.018	-0.040	**0.805**
If I eat something that I would normally not allow myself to eat, I feel pleasure	0.009	-0.027	-0.028	0.002	**0.794**
If I eat something that I would normally not allow myself to eat, I feel satisfied	-0.005	0.009	0.020	0.073	**0.772**

Factor labels were generated to reflect the main theme of each of the factors in the final solution. Factor 1, ‘Strict Behaviours’, contains items that reflect a rigid pattern of behaviour, including following strict rules for one’s food intake and expending significant time and energy towards dietary control. Factor 2, ‘Negative Emotions’, includes items about the experience of negative emotions such as guilt when an individual eats something that they would normally not allow themselves to eat. Factor 3, ‘Worry’, contains items that reflect concerns around the loss of control of food intake. Factor 4, ‘Flexible Beliefs’, contains items that are indicative of a more flexible cognitive approach to dieting, including the belief that any food is acceptable to consume occasionally and in small amounts. Factor 5, ‘Positive Emotions’, contains items that describe the experience of positive emotions such as delight when an individual eats something that they would normally not allow themselves to eat. Based on the definitions of rigid and flexible control that were used to generate items for this new measure, it would appear that higher scores on the Strict Behaviours, Negative Emotions, and Worry factors can be considered indicative of a rigid style of dietary restraint, whereas higher scores on the Flexible Beliefs and Positive Emotions factors can be considered indicative of a flexible style of dietary restraint.

#### Internal consistency and correlations

In this sample, all five factors were found to have good-to-excellent internal consistency [53]. See Table 1 for internal consistency coefficients. The factors that align with a rigid style of control (Strict Behaviours, Negative Emotions, and Worry) correlated positively with one another, and the factors that align with a flexible style of control (Flexible Beliefs and Positive Emotions) correlated positively with one another. The Flexible Beliefs and Positive Emotions factors were both negatively correlated with the Strict Behaviours, Negative Emotions, and Worry factors. See Table 2 for the correlation coefficients.

**Table 2:** Bivariate correlations between the five factors of the FORCES

	RC: Negative Emotions	RC: Worry	FC: Flexible Beliefs	FC: Positive Emotions
RC: Strict Behaviours	.441*	.426*	-.263*	-.187*
RC: Negative Emotions		.664*	-.326*	-.259*
RC: Worry			-.215*	-.173*
FC: Flexible Beliefs				.335*

*RC* Rigid Control, *FC* Flexible Control

^*^ *p* < .001

### Discussion

The aim of Study 1 was to develop a new scale that provided a clearer separation between the rigid and flexible control of eating than the existing measures. Exploratory factor analysis resulted in the FORCES, a 19-item scale with 5 subscales. All five subscales demonstrated good internal consistency in this sample of women. Three of the subscales (Strict Behaviours, Negative Emotions, and Worry) are conceptually aligned with a rigid style of control and are negatively correlated with the other two subscales (Flexible Beliefs and Positive Emotions) which are conceptually aligned with a flexible style of control. This pattern of associations suggests a clear separation between the concepts of rigid and flexible control, and therefore a significant improvement on the previous measures. The aim of Study 2 was to replicate the 5-factor structure in a new sample of women using confirmatory factor analysis. The statistical appropriateness of classifying the factors into higher order groupings of rigid and flexible control was also examined.

## Study 2

### Method

#### Participants

The recommended sample size for confirmatory factor analysis using structural equation modelling is a ratio of 10:1 for participants to the number of parameters to be estimated [30]. The current model included 29 parameters (19 factor loadings and 10 covariances between factors). Exceeding this recommendation, 302 female participants were recruited through Prolific to complete the study online. Participants were recompensed 0.75GBP for completing the 5-min survey. The same eligibility criteria applied as that in Study 1. From sample characteristic information provided during the survey, one participant self-identified as male and was therefore removed from the dataset. Two additional participants were removed from the sample after failing data quality checks, leaving 299 participants for analysis. Of the remaining participants, 296 identified as female, 2 as non-binary, and 1 preferred not to say. Participants had a mean age of 34.54 years (*SD* = 12.46, range 18–87), and a mean BMI of 27.46 (*SD* = 10.19, range 11.29–133.31). In terms of ethnicity, 67.2% of participants identified as White, 18.1% as Asian, 5.4% as Black/African American, 3.3% as Hispanic or Latino(a), and the remaining 6% selected ‘other’ or did not respond. When asked if they had ever been diagnosed with a clinical eating disorder, 4.0% of the sample indicated that they had, 95.3% had not, and 0.7% preferred not to say.

#### Materials and procedure

The materials and procedure used were the same as in Study 1, except that the participants completed the shortened 19-item version of the FORCES. This study received ethics approval from the UNSW HREAP-C (File 3654).

#### Analysis

To determine whether the five-factor structure from Study 1 was replicated in this sample, confirmatory factor analysis was conducted using structural equation modelling with maximum likelihood estimation. The factor loadings for each item were examined, as well as a series of model fit statistics. Good model fit is typically indicated by a non-significant chi-squared test (although this test is sensitive to sample size), a ratio of chi-square to degrees of freedom < 2, comparative fit index (CFI) > 0.95, Tucker-Lewis index (TLI) > 0.95, standardised root mean squared residual (SRMR) < 0.08, root mean square error of approximation (RMSEA) < 0.06 (with upper CI < 0.08), and incremental fit index (IFI) > 0.95 [57–59]. In addition, the modification indices for the regression weights and covariances were examined to ensure they were below 50 and 80, respectively [59]. A secondary analysis was run in which the five factors were loaded onto two higher-order factors representing rigid and flexible control, and the model fit statistics were again examined. Specifically, the Strict Behaviours, Negative Emotions, and Worry factors were loaded onto a Rigid Control factor, and the Flexible Beliefs and Positive Emotions factors were loaded onto a Flexible Control factor. Analyses were conducted using SPSS Amos Version 28.0.0 [60] and SPSS version 28.0.1.1 (14) [55].

### Results

#### Confirmatory factor analysis

Across the 19 items on the FORCES, there were no missing data, and all items had sufficient variation in responses. One item was positively skewed, but the skewness value fell only slightly above the recommended cut-off of 2 (skewness statistic = 2.26), so this item was not transformed. All other items were normally distributed.

Each of the factor loadings were statistically significant and ranged from 0.60 to 0.93 (see Figure S1). Examination of the model fit statistics indicated that the model fit the data very well [57–59]. The chi-squared test was significant (*χ*^2^(142) = 240.52, *p* < 0.001), but the ratio of chi-square to degrees of freedom was 1.69. The other model fit indices also suggested a very good fit: CFI = 0.97, TLI = 0.97, SRMR = 0.05, RMSEA = 0.05 (90% CI [0.04, 0.06]), and IFI = 0.97. In addition, the modification indices for the regression weights and covariances were below 50 and 80, respectively. A secondary analysis in which the five factors were loaded onto higher order rigid and flexible control factors also found that the model fit the data well. Specifically, the overall chi-squared test was significant (*χ*^2^(146) = 254.66, *p* < 0.001), but the ratio of chi-square to degrees of freedom was 1.74, CFI = 0.97, TLI = 0.96, SRMR = 0.06, RMSEA = 0.05 (90% CI [0.04, 0.06]), and IFI = 0.97.

#### Internal consistency

In this sample, the reliability of the five factors ranged from good to excellent [53]. Coefficient alpha and McDonald’s omega indicated that the Strict Behaviours (α = 0.83, ω = 0.84), Worry (α = 0.88, ω = 0.89), Flexible Beliefs (α = 0.81, ω = 0.82), and Positive Emotions (α = 0.88, ω = 0.88) factors demonstrated evidence of good reliability in this sample of women, and the Negative Emotions factor (α = 0.94, ω = 0.94) demonstrated evidence of excellent reliability in this sample.

#### Correlations between factors

As in Study 1, the three rigid control factors (Strict Behaviours, Negative Emotions, and Worry) were positively correlated with one another, and the two flexible control factors (Flexible Beliefs and Positive Emotions) were positively correlated with one another. The rigid control factors were either negatively correlated or had no association with the flexible control factors. The correlation coefficients are displayed in Table 3.

**Table 3:** Bivariate correlations between the five factors of the FORCES in study 2

	RC: Negative Emotions	RC: Worry	FC: Flexible Beliefs	FC: Positive Emotions
RC: Strict Behaviours	.385***	.330***	-.135*	-.164**
RC: Negative Emotions		.590***	-.064	-.218***
RC: Worry			-.047	-.027
FC: Flexible Beliefs				.359***

*RC* Rigid Control, *FC* Flexible Control

^*^ *p* < .05

^**^ *p* < .01

^***^ *p* < .001

### Discussion

Study 2 replicated the five-factor structure of the FORCES from Study 1 in a new female sample. Confirmatory factor analysis demonstrated that this model fit the data well, and a secondary analysis identified that a model in which the subscales were loaded onto higher order factors for rigid and flexible control also fit the data well. As in Study 1, all five factors had good internal consistency. The pattern of correlations between factors suggests that the FORCES clearly separates the measurement of rigid and flexible control and addresses the criticism that scores on the existing measures were often positively correlated with one another [24–26]. In Study 3, the aim was to establish the convergent and discriminant validity of the FORCES for use in female samples by examining the associations between the newly developed scale and measures of dietary restraint, disinhibition, and broader thinking patterns such as dichotomous thinking.

## Study 3

### Method

#### Participants

Participants were 300 females recruited via Prolific, exceeding the 200 participants needed to detect a small-to-medium effect size in a correlation analysis. Participants were recompensed 2.4GBP for completing the 16-min survey. The same eligibility criteria applied as those used in Studies 1 and 2. In sample characteristic questions completed during the study, one respondent self-identified as male and was subsequently removed from the dataset, leaving 299 participants for analysis. Of the 299 participants, 297 identified as female, 1 as non-binary and 1 indicated that they used a different term to describe their gender. The same data quality checks were included as the previous studies, and all participants completed them correctly. Participants had a mean age of 35.3 years (*SD* = 12.9, range 18–74), and the mean BMI was 26.96 (*SD* = 8.14, range 7.96–56.08). In the sample, 69.9% identified their ethnicity as White, 13% as Asian, 7.7% as Black/African American, 5.4% as Hispanic/Latino(a), and 4% as ‘other’. When asked if they had ever been diagnosed with a clinical eating disorder, 6.7% of participants indicated that they had, 91.6% had not, and the remaining 1.7% preferred not to say.

#### Materials and procedure

Participants signed up for a study investigating ‘Health Beliefs and Behaviours’ and were informed that they would be asked to complete a series of questionnaires online. After providing electronic consent, participants completed the 19-item FORCES. Participants were then asked to respond to a series of validation measures outlined below (presented in a random order) and completed the same sample characteristic questions as those included in the previous two studies. This study received ethics approval from the UNSW HREAP-C (File 3654).

##### Original rigid and flexible control scales

The existing Rigid and Flexible Control scales [17], originally developed from the TFEQ-Restraint scale, were included as validation measures to examine how the newly developed FORCES compared to existing measures of the same construct. In the existing 16 item measure, rigid control is defined as an ‘all or nothing’ approach to dieting, whereby certain foods are avoided entirely [15]. In the current sample, Cronbach’s alpha for this measure was 0.82 and McDonald’s omega was 0.82 indicating good reliability. Because the existing measure of rigid control incorporated behavioural and emotions items, a positive correlation was expected with the Strict Behaviours and Negative Emotions factors of the FORCES. Based on the conceptual definition of rigid control associated with this measure, scores were expected to correlate negatively with the Flexible Beliefs factor of the FORCES.

In the existing measure, flexible control is defined as a forgiving dieting approach and is assessed using 12 items [17]. Internal consistency of the existing Flexible Control scale was good in the current sample (α = 0.83, ω = 0.83). Based on the conceptual distinction between rigid and flexible control, it was expected that scores on the existing Flexible Control measure would correlate positively with the Flexible Beliefs factor of the FORCES, and negatively with the Strict Behaviours and Negative Emotions factors.

##### Restraint scale

The Restraint Scale [38] is a 10-item measure that assesses the prevalence of dieting concerns and weight fluctuations and is commonly used to identify restrained eaters. In this sample, the measure had good internal consistency (α = 0.82, ω = 0.83). The Restraint Scale identifies individuals who intend to restrict their food intake but often engage in periods of disinhibited eating. Based on the intention to restrict component, it was hypothesised that the Restraint Scale would have a positive correlation with the Strict Behaviours factor, and a negative correlation with the Flexible Beliefs factor. Because restrained eaters identified by the Restraint Scale tend to abandon their restriction and overindulge, and overindulgence is associated with negative affect [61], a positive correlation was expected with the Negative Emotions factor.

##### Three-factor eating questionnaire

In contrast to the Restraint Scale, the TFEQ separates restraint and disinhibition into distinct subscales [16]. The TFEQ-Restraint subscale is comprised of 21 items assessing the degree to which individuals place cognitive restrictions on their food intake. Cronbach’s alpha was 0.90 and McDonald’s omega was 0.90 in this sample, indicating excellent reliability. Given that the TFEQ-Restraint subscale captures the conscious control of food intake and refers to guilt after overeating, positive correlations were expected with the Strict Behaviours and Negative Emotions factors of the FORCES, whereas a negative correlation was expected with the Flexible Beliefs factor.

The TFEQ-Disinhibition subscale includes 16 items assessing an individuals’ susceptibility to overeating in various situations, such as social settings and during different emotional states. Internal consistency for this subscale was good (α = 0.86, ω = 0.86). The original conceptualisation of rigid and flexible control posited that rigid control was associated with high disinhibition, and flexible control was associated with low disinhibition [15, 17]. Therefore, it was expected that the three rigid control factors of the FORCES (Strict Behaviours, Negative Emotions, and Worry) would correlate positively with TFEQ-Disinhibition, and the two flexible control factors (Flexible Beliefs and Positive Emotions) would correlate negatively with TFEQ-Disinhibition.

##### Dichotomous thinking inventory

The Dichotomous Thinking Inventory (DTI) [62] is a 15-item measure assessing an individuals’ tendency to engage in black and white thinking across a broad range of contexts. The measure had excellent reliability in this sample (α = 0.90, ω = 0.90). It was hypothesised that individuals who engage in dichotomous thinking likely also apply this approach to their dieting behaviours, so a positive correlation was expected between the DTI and the Strict Behaviours factor. In contrast, individuals who scored highly on the DTI were expected to have low scores on the Flexible Beliefs factor.

##### Dichotomous thinking in eating disorders scale

The DTEDS [42] is an 11-item measure with two subscales, assessing the tendency to engage in a black and white thinking style both generally about oneself, and specifically for food. Both subscales had good reliability in this sample (α_General_ = 0.88, ω_General_ = 0.89, α_Food_ = 0.86, ω_Food_ = 0.86). Similar to the DTI, it was expected that the DTEDS would correlate positively with the Strict Behaviours factor, and negatively with the Flexible Beliefs factor. No specific hypotheses were made about the general vs. food subscales of the DTEDS.

##### Body mass index

BMI is a value commonly used to reflect the weight status of an individual. Previous studies using the existing measures of rigid and flexible control have shown that rigid control was associated with a higher BMI and flexible control was associated with a lower BMI [17, 21]. However, other studies have failed to fully replicate the relationship between flexible control and a low BMI [26, 29]. Because participants in the current study provided their weight and height in the sample characteristics section of the questionnaire, we conducted an exploratory analysis to examine the association between BMI values and the five factors of the FORCES. There were no specific hypotheses set.

#### Analysis

Structural equation modelling with maximum likelihood estimation was used to confirm the five-factor structure of the newly developed FORCES within another female sample, using the same model fit criteria as in Study 2. The secondary analysis in which the five subscales were loaded onto higher order factors for rigid and flexible control was also used to confirm the appropriateness of classifying the subscales in this way. These analyses were conducted with SPSS Amos Version 28.0.0 [60]. To establish the convergent and discriminant validity of the FORCES, bivariate correlations between the validation measures and each of the factors were examined. This analysis was conducted using SPSS version 28.0.1.1 (14) [55].

### Results

#### Confirmatory factor analysis

In this study, there were no missing data across the 19 items of the FORCES. Responses on each item were normally distributed. The factor loadings were significant, ranging from 0.56 to 0.94 (see Figure S2), and the model again fit the data very well. The chi-squared test of model fit was significant (*χ*^2^(142) = 235.12, *p* < 0.001), but the ratio of degrees to freedom to the chi-squared value was 1.66, indicating good overall fit. The fit indices also suggested a good fit: the CFI = 0.98, TLI = 0.97, SRMR = 0.04, RMSEA = 0.05 (90% CI [0.04, 0.06]), and IFI = 0.98. The modification indices for the regression weights and covariances were below the recommended cut-offs of 50 and 80, respectively. The model in which the five subscales were loaded onto higher order rigid and flexible control factors also fit the data well in this sample. Specifically, the overall chi-square test was significant (*χ*^2^(146) = 261.08, *p* < 0.001), but the ratio of chi-square to degrees of freedom was 1.79, CFI = 0.97, TLI = 0.97, SRMR = 0.06, RMSEA = 0.05 (90% CI [0.04, 0.06]), and IFI = 0.97. In sum, the five-factor structure of the FORCES was again confirmed in this sample. The appropriateness of classifying the Strict Behaviours, Negative Emotions, and Worry factors as rigid control, and the Flexible Beliefs and Positive Emotions factors as flexible control was again supported in this sample.

#### Internal consistency

In this sample of women, the reliability of the five factors was good to excellent. Cronbach’s alpha and McDonald’s omega for the Strict Behaviours (α = 0.84, ω = 0.84), Flexible Beliefs (α = 0.86, ω = 0.87), and Positive Emotions (α = 0.87, ω = 0.87) factors indicated good reliability in this sample. The Negative Emotions (α = 0.95, ω = 0.95) and Worry (α = 0.90, ω = 0.91) factors demonstrated evidence of excellent reliability in this sample.

#### Correlations between factors

Bivariate correlations between the five factors in this study are displayed in Table 4. The three rigid control factors (Strict Behaviours, Negative Emotions, and Worry) were positively correlated with one another, as were the two flexible control factors (Flexible Beliefs and Positive Emotions). As in Study 2, the rigid control factors either correlated negatively, or had no association, with the flexible control factors.

**Table 4:** Bivariate correlations between the five factors of the FORCES in study 3

	RC: Negative Emotions	RC: Worry	FC: Flexible Beliefs	FC: Positive Emotions
RC: Strict Behaviours	.497**	.429**	-.255**	-.193**
RC: Negative Emotions		.740**	-.179*	-.370**
RC: Worry			-.102	-.162*
FC: Flexible Beliefs				.392**

*RC* Rigid Control, *FC* Flexible Control

^*^ *p* < .05

^**^ *p* < .01

^***^ *p* < .001

#### Construct validity

See Table 5 for correlation values between the FORCES and each of the validation measures.

**Table 5:** Correlations between the subscales of the FORCES and the validation measures

	FORCES Subscale				
	Rigid Control Subscales			Flexible Control Subscales	
	Strict Behaviour	Negative Emotions	Worry	Flexible Beliefs	Positive Emotions
Original Rigid Control	.571**	.539**	.566**	-.231**	-.315**
Original Flexible Control	.542**	.321**	.292**	-.217**	-.250**
Restraint Scale	.472**	.585**	.677**	-.179*	-.206**
TFEQ					
Restraint	.651**	.448**	.420**	-.327**	-.339**
Disinhibition	.184*	.526**	.663**	-.005	-.097
DTI	.153*	.275**	.225**	-.092	.048
DTEDS					
Food	.468**	.722**	.673**	-.193**	-.262**
General	.283**	.485**	.485**	.017	-.033
BMI	-.021	.191***	.349***	-.025	-.010

*TFEQ* Three Factor Eating Questionnaire, *DTI* Dichotomous Thinking Inventory, *DTEDS* Dichotomous Thinking in Eating Disorders Scale, *BMI* Body Mass Index

^*^ *p* < .05

^**^ *p* < .01

^***^ *p* < .001

##### Rigid and flexible control scales

The original measure of Rigid Control was positively associated with the Strict Behaviours, Negative Emotions, and Worry factors of the FORCES and was negatively associated with the Flexible Beliefs and Positive Emotions factors.

Contrary to hypotheses and the conceptual distinction between rigid and flexible control, the original measure of Flexible Control was *positively* correlated with the rigid subscales of the FORCES (Strict Behaviours, Negative Emotions, and Worry), and *negatively* correlated with the flexible subscales (Flexible Beliefs and Positive Emotions).

##### Restraint scale

The Restraint Scale was positively correlated with the rigid control factors of the FORCES, and negatively correlated with the flexible control factors. Specifically, scores on the Restraint Scale were positively correlated with scores on the Strict Behaviours, Negative Emotions, and Worry factors, and negatively correlated with scores on the Flexible Beliefs and Positive Emotions factors.

##### Three-factor eating questionnaire

TFEQ-Restraint scores were positively correlated with the Strict Behaviours, Negative Emotions, and Worry factors of the FORCES, and negatively correlated with Flexible Beliefs and Positive Emotions. Overall, TFEQ-Restraint scores were positively correlated with the three rigid control factors of the FORCES, and negatively correlated with the two flexible control factors.

TFEQ-Disinhibition scores were also positively correlated with the three rigid control factors of the FORCES (Strict Behaviours, Negative Emotions, and Worry) as expected. Contrary to prediction, the correlations between the TFEQ-Disinhibition and the Flexible Beliefs and Positive Emotions factors were not significant.

##### Dichotomous thinking inventory

The DTI was correlated positively with the Strict Behaviours, Negative Emotions, and Worry factors. Despite an expected negative correlation between the DTI and the Flexible Beliefs factor, the DTI had no significant association with either of the flexible factors of the FORCES (Flexible Beliefs and Positive Emotions).

##### Dichotomous thinking in eating disorders scale

Both the food and general subscales of the DTEDS were found to correlate positively with the Strict Behaviours, Negative Emotions, and Worry factors. Partially supporting the hypothesis, the food subscale was negatively correlated with the Flexible Beliefs factor, but the general subscale had no significant association. The same pattern occurred for the Positive Emotions factor: there was a negative correlation between the food subscale and the Positive Emotions factor, but there was no significant association for the general subscale.

##### Body mass index

Two of the three rigid control factors (Negative Emotions and Worry) were positively correlated with BMI, whereas the Strict Behaviours factor and the two flexible control factors (Flexible Beliefs and Positive Emotions) were not significantly associated with BMI.

### Discussion

In Study 3, the five-factor structure of the FORCES was once again replicated in a female sample, with confirmatory factor analysis suggesting the model fit the data well. As in the previous studies, all five factors had good internal consistency, and the correlations between the factors suggested a clear separation of the concepts of rigid and flexible control. Finally, the rigid control factors were positively correlated with related concepts, whereas the flexible control factors were either negatively correlated or had no significant association, suggesting that the FORCES has good construct validity among women, both in terms of convergent and discriminant validity.

## General discussion

Repeated on–off dieting behaviour can have negative psychological consequences, and is an important target for intervention, particularly among women [2]. To determine how best to target this behaviour, it is important to understand individual variability in the degree to which restrained eaters engage in disinhibited eating. One distinction that may help account for this variability is whether dietary restraint is characterised as either rigid or flexible. However, the existing measures of rigid and flexible control fail to clearly separate the two approaches to dietary restraint. Studies with either predominantly or entirely female samples have identified a positive correlation between the existing measures of rigid and flexible control and have also found that the two subscales correlated in the same direction with other relevant outcomes [24–26]. Thus, the aim of the current studies was to develop a new measure that could more clearly differentiate the rigid and flexible control of food intake. Exploratory and confirmatory factor analysis resulted in the FORCES, a 19-item measure with three factors assessing rigid control (Strict Behaviours, Negative Emotions, and Worry) and two factors assessing flexible control (Flexible Beliefs and Positive Emotions). Across three studies with female participants, the five-factor structure was replicated and validated, and the final scale was shown to have good internal consistency. The three rigid control factors of the FORCES either correlated negatively, or had no association, with the two flexible control factors. The three rigid control factors also had different patterns of association than the two flexible control factors with relevant measures of restraint, disinhibition, and dichotomous thinking, as well as participants BMI. Notably, the original measures of both rigid and flexible control were positively correlated with the FORCES rigid subscales, and were negatively correlated with the FORCES flexible control subscales, further demonstrating the limitations of the original scales. The newly developed scale provides a much clearer separation between the concepts of rigid and flexible control than did the original measures and shows promise at being more useful for predicting the eating-related thoughts and behaviours of women.

### Rigid control factors

In the current study, each of the three rigid control factors positively correlated with the Restraint Scale (known to capture the tendency to vacillate between restriction and overindulgence), and both the Restraint and Disinhibition subscales of the TFEQ. These factors were also found to be reflective of a dichotomous thinking style, positively associated with the DTI and the general and food subscales of the DTEDS. Interestingly, the Strict Behaviours factor was not significantly associated with participants BMI, suggesting that engaging in strict dieting behaviours may not have any (cross-sectional) relation to participants’ weight. However, the Negative Emotions and Worry subscales were both positively associated with BMI.

#### Strict behaviours

It has been previously suggested that attempting to strictly control one’s behaviour can be detrimental and lead to an increase in that behaviour [63]. In the domain of eating behaviour, dieters who score highly on the Strict Behaviours subscale may fail to uphold their strict behavioural intentions, and instead engage in periods of overindulgence. The Strict Behaviours subscale may be particularly useful in future research to identify participants who are likely to display a ‘what the hell’ attitude following a diet transgression [13]. It would be interesting to examine whether this subscale explains overindulgent eating in situations where dieters lack the cognitive capacity to implement their strict dieting regime, such as completing tasks under a high cognitive load, or under the influence of alcohol [11, 12].

#### Negative emotions

The Negative Emotions factor aligns with previous findings that restrained eaters report negative affect after an episode of disinhibited eating and attribute this negative affect to their food consumption specifically [61]. The experience of negative emotions has also been shown to *trigger* periods of disinhibited eating [9, 10, 33], potentially creating a vicious cycle between rigid restriction and overindulgence. It would be interesting to examine whether high scores on this factor are associated with a greater likelihood of disinhibited eating in situations where dieters report experiencing negative or dysphoric mood [8–10]. Future research could also explore whether the experience of negative mood following a diet transgression mediates the relationship between dietary restraint and negative psychological consequences such as body dissatisfaction [3, 4].

#### Worry

Rigid rules regarding dietary control are likely more difficult to follow than flexible guidelines, which may explain why the experience of worry is characteristic of a rigid pattern of restraint. Future research could explore whether dieters who score highly on the Worry subscale have lower dieting self-efficacy than those who are less worried about their dietary restraint [64], and how this may explain variability in disinhibited eating following a diet disruption.

### Flexible control factors

Both flexible control subscales correlated negatively with the Restraint Scale and TFEQ-Restraint. There was no significant association with TFEQ-Disinhibition, suggesting that flexible beliefs and positive emotions neither predict nor prevent disinhibited eating, consistent with the findings that these factors were not associated with participants BMI. Perhaps these two factors need to co-occur, such that participants hold flexible beliefs about dieting and have positive emotional responses towards a diet violation to have a protective effect against disinhibited eating. There were no significant relationships between these factors and general dichotomous thinking. There were, however, negative correlations with food specific dichotomous thinking, suggesting a domain specific pattern and demonstrating the discriminant validity of these factors in the construct of dichotomous thinking.

#### Flexible beliefs

Holding flexible beliefs may help restrained eaters view a diet transgression in a forgiving manner. This subscale may be particularly useful in explaining variability in disinhibited eating following the consumption of a high calorie food, and future research could examine the predictive validity of this subscale in determining eating behaviour in preload studies. It would also be interesting to examine whether participants who hold more flexible beliefs about their food intake are more responsive to internal cues for hunger and satiety.

#### Positive emotions

Experiencing positive emotions after a diet transgression suggests that the dieter views the food consumed as a ‘treat’, and something to be enjoyed. Similar to the Flexible Beliefs subscale, it would be interesting to examine the predictive validity of this subscale in preload studies, to determine whether higher scores on the Positive Emotions subscale are associated with less overindulgent eating following a preload. It is also worth exploring whether individuals who experience more positive emotions after eating something that they would normally not allow themselves experience greater food enjoyment more generally.

### Applications and limitations

Using the FORCES to examine the nature of an individual’s restraint may help future research to examine and understand individual differences in the likelihood of engaging in disinhibited eating. When validating the scale in Study 3, the three rigid control factors showed a positive correlation with scores on the TFEQ-Disinhibition measure, whereas the two flexible control factors showed no association. These findings suggest that greater rigid control might increase an individual’s susceptibility to disinhibited eating, but greater flexible control does not necessarily protect against disinhibition. Future research could examine whether an intervention that attempts to reduce rigid control can successfully reduce disinhibited eating, thereby reducing the negative psychological and physical impacts of repeated dieting. The negative health effects of dieting and weight cycling are an increasingly urgent public health issue [2].

The FORCES may also help future research to understand the relationship between dietary control and patterns of weight control. Previous research using the original measures of rigid and flexible control produced conflicting evidence of the relationship between these styles of dietary restraint and BMI. Samples comprised of participants attempting to lose weight found that rigid control was positively associated with BMI, and flexible control was negatively associated [17, 21]. However, general samples of community members or university students found a positive association between rigid control and BMI but failed to fully replicate the association between flexible control and a lower BMI [26, 29]. Findings of the current study, which recruited community members, align with previous research using similar samples. In the current study, two of the three rigid control factors (Negative Emotions and Worry) were positively associated with BMI, whereas the Strict Behaviours factor and the two flexible control factors had no association with BMI. Similar to the findings for disinhibition, this pattern of results suggests that rigid control may increase an individual’s susceptibility to weight management difficulties, but flexible control may not necessarily protect against these difficulties. It is important to note that BMI is an imperfect measure of weight control and provides a snapshot of participants current weight, without reflecting fluctuations in weight over time. Future research could examine whether flexible control is associated with the maintenance of a more stable weight than rigid control, and whether reducing rigid control assists in reducing weight fluctuations.

The role of flexible control in understanding individual differences in the degree to which individuals engage in disinhibited eating may require further exploration. In Study 3, the flexible control factors were either negatively correlated or had no association with the validation measures. Further research could explore the discriminant validity of the Flexible Beliefs and Positive Emotions factors with additional measures, such as the Unconditional Permission to Eat subscale of the Intuitive Eating Scale [40]. If flexible control does not necessarily protect against disinhibition on its own, it is important to identify the outcomes associated with engaging in a flexible style of control, and to examine how these outcomes compare to engaging in no dietary control whatsoever.

The FORCES is a new measure of the rigid and flexible control of eating, which provides a useful and valid way of assessing rigid and flexible control over one’s eating. The five-factor structure of the scale was replicated across three different samples of women, and each of the factors showed good validity with other relevant measures. Note, however, that the FORCES was validated against other self-report measures. Although these measures have been validated and shown to predict behaviour in previous research, it would be useful to examine whether the FORCES itself has predictive validity for objective behavioural outcomes. Specifically, future studies should examine whether the factors of the FORCES can be used to identify and understand differences in actual food intake.

In the current studies, the FORCES examined the nature of dietary restraint in the context of self-imposed restrictions around eating and dieting. Participants were asked not to consider restrictions based on allergies, intolerances, or medical conditions when completing the items on the scale. Excluding health-related motives for dieting is in line with existing conceptualisations of restrained eating in which dieters are motivated to restrict or control their food intake for appearance reasons such as weight loss or maintenance [1]. Research has previously demonstrated that individuals with varying motivations for dieting (such as health vs. appearance motives) differ on important related constructs, such as body dissatisfaction and body image concerns [44, 45]. However, future research could expand the generalisability of the FORCES to include eating restrictions that may be externally imposed or health-related. This expansion would allow for further investigation of the distinction between different dieting motivations or sources of restriction, and the impact of these distinctions on why some dieters are more likely to cycle between periods of restriction and overindulgence than are others.

The current studies were also limited in terms of the characteristics of the samples, meaning the findings may not be entirely generalisable to other populations. First, the current studies were comprised of female samples, because previous research on dietary restraint and disinhibition has been primarily conducted using female samples [6, 8–12, 32–36], as has research on the original rigid and flexible control measures [15, 19–22, 24, 25, 27–29, 37]. Despite being more common among females, dietary restraint and disinhibited eating behaviours have also been identified among males [65, 66]. Future research could expand the generalisability of the FORCES by establishing the validity of the measure across individuals of all genders. Second, the three samples were comprised of mainly participants who identified as White. In a survey of participants from the United States, disordered eating and dieting behaviours were identified as pervasive problems across all racial and ethnic groups [67]. Examining the validity of the FORCES among other ethnicities would help to broaden the potential applications of this new measure. Third, the current study was advertised as investigating ‘Health Beliefs and Behaviours’. Although there was no mention of eating or dieting behaviour in the study advertisement, it is still possible that participants with a particular interest in health more generally opted to sign up for the study. Ultimately, examining the generalisability of the FORCES across various characteristics, such as education levels and socioeconomic status, is important to ensure wide applicability of this measure.

## Conclusion

The current studies describe the development and validation of the FORCES, a 19-item, five-factor scale assessing the rigid and flexible control of eating. The scale has good internal consistency, a reliable factor structure across three samples of women, and clearly separates the concepts of rigid and flexible control. Future research could help to establish the relationship between the subscales of this new measure and differences in eating behaviour. Improving the assessment of the nature of an individual’s restraint will help to provide further clarity on the existence of individual differences in the likelihood of engaging in disinhibited eating and may provide insights that could be used to design effective interventions to address the negative consequences of rigid dieting behaviour.

## Supplementary Information

Supplementary Material 1.

## Data Availability

The datasets used and/or analysed during the current studies are available from the corresponding author on reasonable request.

## Abbreviations

BMI: Body Mass Index
CFI: Comparative Fit Index
CI: Confidence Interval
DTEDS: Dichotomous Thinking in Eating Disorders Scale
DTI: Dichotomous Thinking Inventory
FORCES: Flexible Or Rigid Control of Eating Scale
IFI: Incremental Fit Index
KMO: Kaiser–Meyer–Olkin
MAP: Minimum Average Partial
RMSEA: Root Mean Square Error of Approximation
SD: Standard Deviation
SRMR: Standardised Root Mean Squared Residual
TFEQ: Three Factor Eating Questionnaire
TLI: Tucker-Lewis Index

## Footnotes

1 Note that in all three studies, participant BMI was calculated using their self-reported weight and height and may contain some improbable values. All values as reported by participants have been included in the calculation of these descriptive statistics.
