# Supplementary material for: Flexible or rigid control of eating scale: development and validation of the FORCES in women
Source: Int J Behav Nutr Phys Act. 2025 Apr 11;22:45. doi: 10.1186/s12966-025-01746-3 (PMC11992708; doi:10.1186/s12966-025-01746-3)
Supplement: Supplementary file 1 — Supplementary Material 1. [file 12966_2025_1746_MOESM1_ESM.docx]

**Figure S1.**

*Standardised Regression Weights (Factor Loadings) for the Confirmatory Factor Analysis in Study 2.*


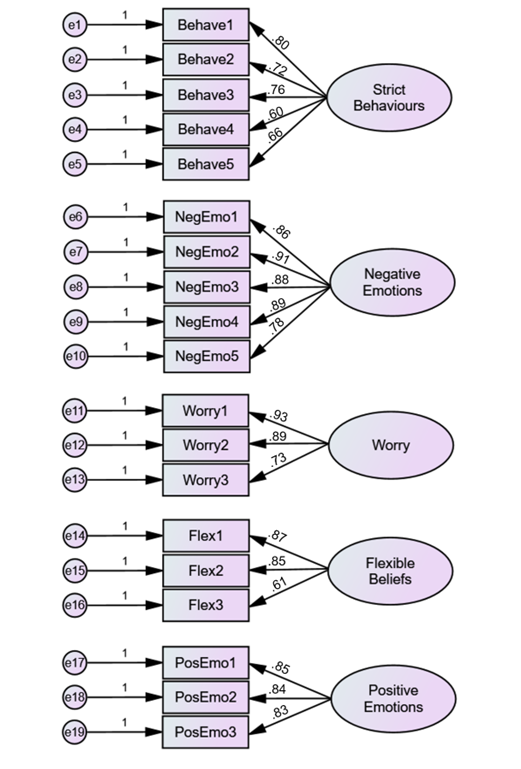


*Note that for Clarity, Covariances Between Factors are Not Displayed.*

**Figure S2.**

*Standardised Regression Weights (Factor Loadings) for the Confirmatory Factor Analysis in Study 3.*


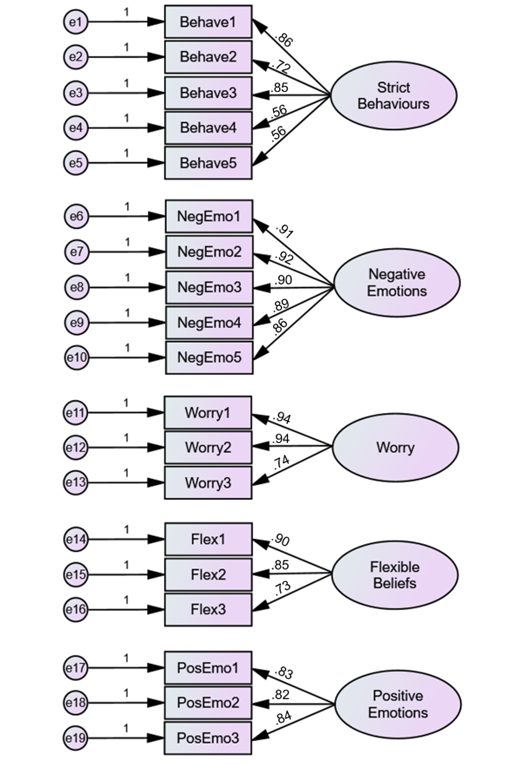


*Note that for Clarity, Covariances Between Factors are Not Displayed.*
